# Upscaled Production of Satellite-Free Droplets: Step Emulsification with Deterministic Lateral Displacement

**DOI:** 10.3390/mi15070908

**Published:** 2024-07-12

**Authors:** Guangchong Ji, Shuzo Masui, Yusuke Kanno, Takasi Nisisako

**Affiliations:** 1 Department of Mechanical Engineering, School of Engineering, Tokyo Institute of Technology, Tokyo 152-8550, Japan; ji.g.aa@m.titech.ac.jp; 2 Institute of Innovative Research, Tokyo Institute of Technology, R2-9, 4259 Nagatsuta-cho, Midori-ku, Yokohama, Kanagawa 226-8503, Japan; masui.s@nanolab.t.u-tokyo.ac.jp (S.M.); kanno.y.ag@m.titech.ac.jp (Y.K.)

**Keywords:** step emulsification, deterministic lateral displacement, parallelization, droplet separation, satellite droplets

## Abstract

Step emulsification is a key technique for achieving scalable production of monodisperse emulsion droplets owing to its resilience to flow fluctuations. However, the persistent issue of satellite droplets, an inherent byproduct of main droplets, poses challenges for achieving truly uniform product sizes. In a previous study, we introduced a module with step-emulsifier nozzles upstream and deterministic lateral displacement (DLD) micropillar arrays downstream to generate satellite-free droplets at a low throughput. In this study, we demonstrate an upscaled parallelized setup with ten modules that were designed to produce satellite-free droplets. Each module integrated 100 step-emulsification nozzles in the upstream region with DLD micropillar arrays downstream. We conducted 3D flow simulations to ensure homogeneous distribution of the input fluids. Uniformly supplying an aqueous polyvinyl alcohol solution and an acrylate monomer as continuous and dispersed phases into the ten modules, the nozzles in each module exhibited a production rate of 539.5 ± 28.6 drop/s (*n* = 10). We successfully isolated the main droplets with a mean diameter of 66 μm and a coefficient of variation of 3.1% from satellite droplets with a mean diameter of 3 μm. The total throughput was 3.0 mL/h. The high yield and contamination-free features of our approach are promising for diverse industrial applications.

## 1. Introduction

Step emulsification, also known as microchannel emulsification [1], is a prominent microfluidic method for generating monodisperse emulsion droplets [2,3]. In this method, droplets form at a step created by a nozzle terminating in a deep main channel. Unlike droplet formation via shear stress, which is common in other techniques [4,5,6,7,8], step emulsification depends on interfacial tension influenced primarily by nozzle geometry [9] and surface wetting [10]. Consequently, the size of droplets produced in the dripping regime in step emulsification does not depend on the flow rates of the continuous and dispersed phases; instead, it is determined by the geometry of the nozzle, particularly its height. This resistance to changes in flow rates makes it highly advantageous for scaled-up production, especially in applications involving parallel nozzles.

Previous studies have demonstrated various configurations of parallel step-emulsifier nozzles to produce monodisperse emulsion droplets. A common configuration is the “cross-flow” setup, where a continuous phase flows vertically across an array of parallel nozzles to collect the produced droplets [11]. For instance, Amstad et al. developed a “millipede” configuration with 550 parallel, wedge-shaped step-emulsifier nozzles made from polydimethylsiloxane (PDMS), successfully producing monodisperse water-in-oil droplets [9]. Additionally, a silicon-based device with dry-etched “straight-through” nozzles is noted for its high-density parallelization [12,13]. We have also recently reported a vertical slit configuration for nozzle parallelization to collect droplets without coalescence [14]. 

Devices with parallel step emulsifiers have been effectively used to produce various functional microparticles [15]. For instance, de Rutte et al. created monodisperse bioactive microgel particles using a PDMS device with an array of 200 nozzles, achieving throughputs of 7.2 mL/h and 14.4 mL/h [16]. Additionally, the production of complex double emulsions using parallel step emulsifiers has been reported [17,18].

Despite its benefits, step emulsification often results in the formation of undesirable satellite droplets due to Plateau–Rayleigh instability, a challenge that has received limited attention. Recently, several studies have reported the continuous separation of main and satellite droplets through bifurcation channels arranged after T-junction or flow-focusing emulsifiers [19,20,21]. Also, deterministic lateral displacement (DLD) [22,23], a size-based separation method, was coupled with a flow-focusing droplet maker to produce satellite-free droplets with a throughput of up to 0.2 mL/h [24,25]. In our earlier study, we designed a module that coupled 60 parallel step-emulsification nozzles and DLD micropillar arrays and could produce satellite-free droplets [26]. However, this device exhibited a low throughput of 0.2 mL/h, necessitating the upscaled production via an efficient module parallelization strategy.

In this study, we propose an integrated system comprising ten parallel-arranged modules coupled with step emulsification and DLD that demonstrated effective production of satellite-free droplets. Through 3D flow simulations, we demonstrated the fluid distribution in the top part. Experiments showed the formation of main droplets with narrow size distributions and effective size-based separation of the main and satellite droplets via DLD arrays in the ten modules.

## 2. Materials and Methods

### 2.1. Device Design and Mechanism

**Figure 1:**
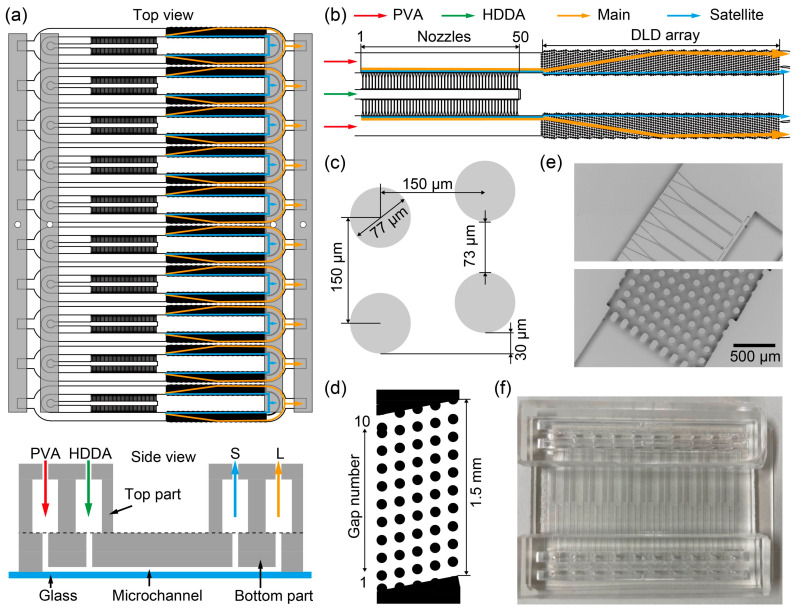
A microfluidic device consisting of ten parallelized step-emulsification and deterministic lateral displacement (DLD) modules for separating main and satellite droplets. (**a**) Schematic illustration of the top and cross-sectional side views of the parallelized device. (**b**) Detailed schematic of an individual module. (**c**) A rhombic unit of the DLD pillars. (**d**) A single region containing the five-column DLD pillars. (**e**) SEM images showing the step-emulsifier nozzles and the DLD array. (**f**) Photograph of the polydimethylsiloxane (PDMS) device sealed by a 72 mm × 56 mm glass slide.

We designed a microfluidic device with two integral sections: An upper segment featuring four chambers for liquid introduction and droplet collection and a lower segment housing ten modules equipped with step-emulsification nozzles and DLD micropillar arrays for droplet production and separation (Figure 1a). The sections were assembled seamlessly and covered with a glass slide. Each of the top chambers was 60 mm long, 3 mm wide, and 5 mm high, with a central aperture for fluid input or droplet collection. The bottom part comprised ten parallel modules, each integrating a step-emulsification nozzle and a DLD micropillar array. Each module had two inlets for the dispersed and continuous phases and two outlets for the main and satellite droplet collections, which were serially aligned. The inlets and outlets were interconnected using dedicated chambers. Each module had a symmetric layout with a central channel for the dispersed phase flanked by two arrays of 50 step-emulsification nozzles (i.e., 100 nozzles in total). Two periodic arrays of DLD micropillars are positioned downstream of the main channel, linking separate outlets for collecting the main and satellite droplets (Figure 1b). 

The step-emulsifier nozzles, aligned in parallel with a 200 μm pitch and dimensions of 1 mm in length and 18 μm in depth, facilitate fluid transfer between the central and main channels of 80 μm in depth (Figure S1a–c). Each nozzle features a wedge-shaped end that opens toward the main channel, with the width expanding from 25 to 140 μm over a length of 333 μm at a 9.8° angle (Figure S1d).

Downstream, the arrayed nozzles were followed by periodically arrayed DLD micropillars, with each rhombic unit cell comprising four micropillars 77 μm in diameter and spaced 150 μm apart, yielding a gap of 73 μm between adjacent pillars (Figure 1c). A shift of 30 μm is observed between two adjacent columns of the pillars, and a single DLD region comprises five columns and ten row gaps (Figure 1d). This DLD array comprises 20 repeats of single DLD regions toward the outlets, facilitating the lateral displacement of the main droplets toward the side wall. The critical diameter *D*_c_, representing the cutoff size for particle separation in DLD, is calculated by the following equation [27]:

*D*_c_ = 1.4 × *G* × (Δ*λ*/*λ*)^0.48^ (1)

where *G* represents the gap between pillars (73 μm), *λ* denotes the pitch between pillars (150 μm), and ∆*λ* (30 μm) signifies the shift between pillars. Consequently, the *D*_c_ of our device was 47 μm.

The dispersed and continuous phases were introduced separately from each inlet at the top, which filled the inlet chambers and descended via the ten module inlets, flowing into the lower segment. The dispersed phase subsequently entered the center channels (600 μm wide) and filled the array nozzles. The fluid flow rates were controlled within the dripping regime to facilitate the formation of monodisperse main droplets at the nozzle ends along with satellite droplets as byproducts. These droplets, guided by the continuous phase within the main channel, moved into the DLD region, where main droplets exceeding *D*_c_ in diameter underwent lateral migration at the pillar shift angle (bump mode). Conversely, the satellite droplets followed the average global flow direction (zigzag mode). Following the DLD separation, the main and satellite droplets exited through outlets L and S at the termini of the microchannels, respectively, and were collected by the top chambers.

### 2.2. Device Fabrication

The bottom part was fabricated using PDMS via standard soft lithography. This process involved a two-step fabrication of an SU-8 mold for the nozzles (height: 18 μm) and other regions (height: 80 μm) on a silicon wafer (diameter: 4 in), followed by obtaining a PDMS replica, as previously reported by our research group (Figure 1e) [26].

The top part was cast using PDMS with an acrylic mold. Four rectangular acrylic blocks (5 mm × 60 mm × 3 mm) were cut from an acrylic board using a laser processing machine (Epilog Fusion Pro 36; Tokyo, Japan). These blocks were arranged and affixed to a Petri dish using double-sided tape. Subsequently, holes (diameter: 1 mm) were produced in the two inlets and outlets using a punch tool.

The bottom part was bonded to the top part using oxygen plasma treatment (18 W, 0.3 Torr, 1 min; PDC-32G, Harrick Plasma, NY, USA) and then baked at 80 °C for 2 h on a hot plate (HI-1000; AS ONE, Osaka, Japan). The microchannels in the bottom part were sealed with a glass slide (S9111; Matsunami Glass, Osaka, Japan) using oxygen plasma bonding (18 W, 0.3 Torr, 40 s) (Figure 1f). Immediately after bonding the bottom part to the glass slide, 0.5 mL of an aqueous reagent containing a superhydrophilic polymer (SPRA-202; Tokyo Ohka Kogyo, Kanagawa, Japan) was injected into the device using a 1 mL disposable syringe (Terumo, Tokyo, Japan), rendering the surface of the microchannels hydrophilic. After wetting the surface for 1 min, it was rinsed with pure water (Direct-Q UV3; Merck, Hessen, Germany) and manually dried with a disposable syringe. The device was then baked again at 80 °C for 2 h on a hot plate.

### 2.3. Chemicals

For the continuous phase, a 2 wt% aqueous solution of polyvinyl alcohol (PVA; GL-03; *M*_w_ approximately 20,000 g/mol; 87–89% hydrolyzed; Mitsubishi Chemical, Tokyo, Japan) was prepared. For the dispersed phase, 1,6-hexanediol diacrylate (HDDA; Shin-Nakamura Kagaku, Tokyo, Japan) was used.

### 2.4. Equipment

A 10 mL gastight glass syringe (1010TLL; Hamilton, NV, USA) was used for the HDDA, while a 50 mL disposable syringe (SS-50ESZ; Terumo, Tokyo, Japan) was used for the aqueous PVA solution. Both syringes were equipped with syringe needles (SNA-22G-C; Musashi Engineering, Tokyo, Japan) and connected to the top-part inlets via polyethylene tubes (0.5 mm i.d., 1.0 mm o.d.; Hibiki, Kunii, Tokyo, Japan). Two syringe pumps (Legato 200; KD Scientific, Holliston, MA, USA) were employed to drive the syringes. Droplet formation and migration within the microfluidic device were recorded using an inverted optical microscope (IX 73; Olympus, Tokyo, Japan) equipped with a high-speed video camera (Fastcam Mini AX50; Photron, Tokyo, Japan). 

### 2.5. CFD Simulation

#### 2.5.1. Simulation Setup

Fluid flow simulations were conducted using commercial software (Fluent 19.0; Ansys, Canonsburg, PA, USA) to analyze the flow distributions within the inlet chamber channels and DLD section. The geometries of these channels were constructed using Ansys Meshing, resulting in models with approximately 1 million tetrahedron-shaped grid cells for the inlet chamber and 0.8 million for the DLD section. The maximum face size was set to 200 μm for the inlet chamber and 5 μm for the DLD section to ensure a detailed resolution.

#### 2.5.2. Boundary Conditions and Working Fluid

Stationary and no-slip boundary conditions were applied to the solid channel walls. Specific mass flow rates were set at the inlets, and a pressure–outlet boundary condition was applied with the gauge pressure set to 0 Pa. Water was chosen as the working fluid, with a density of 998.2 kg/m^3^ and a dynamic viscosity of 1.003 mPa s.

#### 2.5.3. Solver Settings and Convergence Criteria

A pressure-based solver for steady-state simulations with a viscous laminar flow model was selected. The SIMPLE algorithm was employed for pressure–velocity coupling. For spatial discretization, a second-order upwind scheme was chosen for momentum equations. Residuals for continuity and momentum equations were monitored, and the solution was considered converged when the residuals became stable below 10^−3^.

## 3. Results and Discussion

### 3.1. Three-Dimensional Fluid Flow Simulation

In step emulsification, droplet size is primarily determined by nozzle geometry rather than flow rates. However, when the dispersed phase flow rate at a nozzle exceeds a threshold, the droplet generation mode shifts from dripping to jetting, causing a significant increase in droplet size [3,9]. Uneven distribution of the dispersed phase flow rate among the modules might force some nozzles to operate in jetting mode, making the product polydisperse [14]. Therefore, uniform supply of the dispersed phase among the modules is important. Achieving this requires uniform pressure distribution among the modules, supported by uniform distribution of the continuous phase flow rate. Thus, to upscale the production of monodisperse droplets, achieving a uniform flow rate distribution across different modules is important, even in step emulsification. 

**Figure 2:**
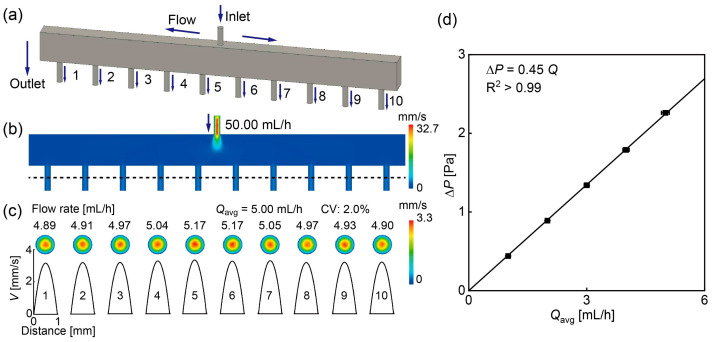
A 3D fluid flow simulation focusing on the inlet reservoir. (**a**) Model depicting the inlet reservoir. (**b**) Velocity distribution across the cross-section of the inlet reservoir. (**c**) Velocity distribution and profile in the middle of the ten outlet pipes. The input flow rate was set at 50.0 mL/h. (**d**) Pressure drop analysis of the inlet chamber across various average outlet flow rates. The calculated average hydraulic resistance *r*_avg_ was 1.62 GPa·s/m^3^.

**Figure 3:**
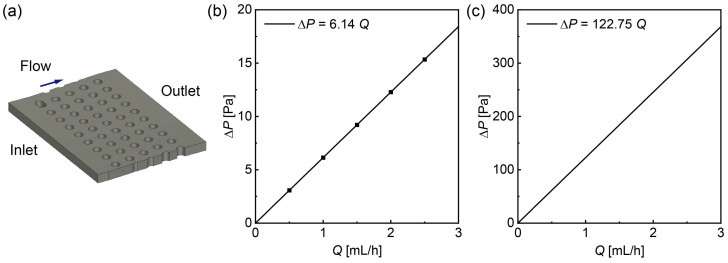
A 3D fluid flow simulation focusing on a single DLD section. (**a**) Model of a single DLD section within the microchannel. (**b**) Pressure drop of the individual DLD section plotted against the flow rate. The calculated hydraulic resistance was 2.21 × 10^1^ GPa·s/m^3^. (**c**) Pressure drop of the DLD array comprising 20 DLD sections versus the flow rate. The calculated hydraulic resistance was 4.42 × 10^2^ GPa·s/m^3^.

To evaluate the flow rate distribution across the ten parallelized modules, a 3D fluid flow simulation of the inlet chamber and a single DLD module was conducted in conjunction with an equivalent electric circuit model, as performing a CFD analysis of the entire setup was computationally expensive. Initially, distinct models of the inlet chamber (Figure 2a) and the DLD pillar array (Figure 3a) were developed to calculate the hydraulic resistance of each part. We used pure water (viscosity: 1 mPa s) as a model fluid in the simulations. Under low Reynolds number conditions, the hydraulic resistance is proportional to the fluid viscosity, and the flow rate distribution, determined by the ratio of the hydraulic resistances, is not affected by fluid viscosity. Subsequently, an equivalent electrical circuit model was used to determine the flow distribution to each DLD module via the top chamber.

The inlet chamber model included a via hole at the top as the inlet (1 mm in diameter, 3 mm long), a straight channel with a rectangular cross-section (60 mm long, 3 mm wide, and 5 mm high), and ten via holes at the bottom as the outlets (1 mm in diameter, 4 mm long; Figure 2a). The flow rate distribution across the ten outlets was initially calculated by applying a flow rate of 50 mL/h of water to the inlet, with the outlets open to the atmosphere. The fluid velocity at the vertical cross-section of the model is shown in Figure 2b, demonstrating low and uniform velocity in the horizontal chamber and at the ten outlets. The flow rate distribution at the ten outlets and fluid velocity profile in the middle of the outlet pipes are shown in Figure 2c, with flow rates averaging 5.00 mL/h with a CV of 2.0%, indicating an even fluid distribution from the inlet chamber. The velocity profiles exhibited the parabolic profile characteristic of Hagen–Poiseuille flow. 

When the inlet flow rate varied from 10 to 50 mL/h, the CVs of the outlet flow rates ranged from 2.0 to 2.4%, demonstrating uniform flow distributions. This result suggests that hydraulic resistances of ten vertical outlet pipes are more dominant than the resistance from horizontal resistor elements in the rectangular chamber. Therefore, the 3D model in Figure 2a can be approximated by an equivalent electric circuit comprising ten parallel resistors (*r*_1–10_) of similar values. 

Figure 2d illustrates the relationship between the average outlet flow rate *Q*_avg_ and the pressure drop ∆*P* when the inlet flow rate varied from 10 to 50 mL/h. As *Q*_avg_ increased from 1.0 to 5.0 mL/h, ∆*P* rose linearly from 0.44 to 2.26 Pa. The linear relationship between ∆*P* and *Q*_avg_ confirms the Hagen–Poiseuille law ∆*P* = *Q*_avg_*R*, where *R* is the hydraulic resistance. The hydraulic resistance for the ten outlets (*r*_1–10_), calculated from the pressure drop over each outlet flow rate, ranged from 1.57 to 1.66 GPa·s/m^3^, with an average hydraulic resistance *r*_avg_ of 1.62 GPa·s/m^3^.

The hydraulic resistance of a DLD section with five columns and ten rows (Figure 3a) was also calculated similarly. Flow rates ranging from 0.5 to 2.5 mL/h were applied to the inlet of a single DLD section, with the outlet open to the atmosphere. This resulted in a linear increase in pressure drop ∆*P* from 3.07 to 15.34 Pa as the flow rate increased. The hydraulic resistance of a single DLD section *R*_0_ was determined to be 2.21 × 10^1^ GPa·s/m^3^ (Figure 3b). For a DLD micropillar array consisting of 20 serial repeats of the single DLD section, the hydraulic resistance *R*_D_ was calculated to be 4.42 × 10^2^ GPa·s/m^3^, twenty times that of the single DLD section *R*_0_ (Figure 3c). 

The hydraulic resistance of the microfluidic channel without pillars *R*_c_ was calculated using the following equation [28]:

(2) $$R_{c}=\frac{12\eta L}{h^{3}w}{\left(1-\frac{192h}{\pi^{5}w}\right)}^{-1}$$

where *η* is the fluid viscosity, *L* is the length of the channel, and *w* and *h* are the width and height of the rectangular channel, with *w* > *h*. Substituting *η* = 1 mPa s, *L* = 17.8 mm, *w* = 1.275 mm, and *h* = 80 μm, we obtained *R*_c_ = 3.41 × 10^1^ GPa s/m^3^.

To determine the flow rate distribution across the ten modules, we used a simplified model treating the top chambers and the ten bottom modules as an equivalent parallel electronic circuit (Figure S2). For simplicity, the channels supplying the dispersed phase were neglected due to their low volume fraction (5.7%). The channels downstream of the DLD region, with two bifurcating outlet chambers, were simplified as one outlet with half the hydraulic resistance of inlet chamber *r*_1–10_. The hydraulic resistance of the ten modules, which share the same inlet and outlet, was modeled in a parallel arrangement. Thus, for each module, the inlet chamber (*r*_1–10_), the microfluidic channel with and without DLD pillars (0.5*R*_c_ + 0.5*R*_D_), and the outlet chamber (0.5*r*_1–10_) were arranged in series. The ratio of flow rates of *m*-th and *n*-th DLD modules was calculated using the following equation:

(3) $$\frac{Q_{m}}{Q_{n}}=\frac{r_{n}+{0.5R_{c}+0.5R}_{D}+0.5r_{n}}{r_{m}+{0.5R_{c}+0.5R}_{D}+0.5r_{m}}=\frac{1+\frac{3r_{n}}{{R_{c}+R}_{D}}}{1+\frac{{3r}_{m}}{{R_{c}+R}_{D}}}$$

where *Q_m_* and *Q_n_* are the flow rates out of module *m* and module *n*, respectively. Because the hydraulic resistance of the inlet chamber was significantly lower than that of the microfluidic channel with and without DLD micropillars (*R*_c_ + *R*_D_ = 294 *r*_avg_), the calculated flow rate ratio *Q_n_*/*Q*_1_ showed a minor variation of less than 0.06% among the ten modules (Table S1). Therefore, we confirmed that uniform flow distribution can be achieved for each module by combining numerical simulation and theoretical models.

### 3.2. Step Emulsification in Parallelized Modules

In this section, we describe the process of droplet generation using arrayed step-emulsification nozzles within each module. We used an aqueous PVA solution as the continuous phase and HDDA as the dispersed phase to compare the performance of the proposed device with the single-module device in our previous study [26], which used the same materials. Following confirmation of the main and satellite droplet formation at the step-emulsification nozzles across the ten modules, we conducted measurements to compare the droplet sizes and generation rates within each module. Additionally, we calculated and assessed the flow rates of the dispersed phases across different modules. 

**Figure 4:**
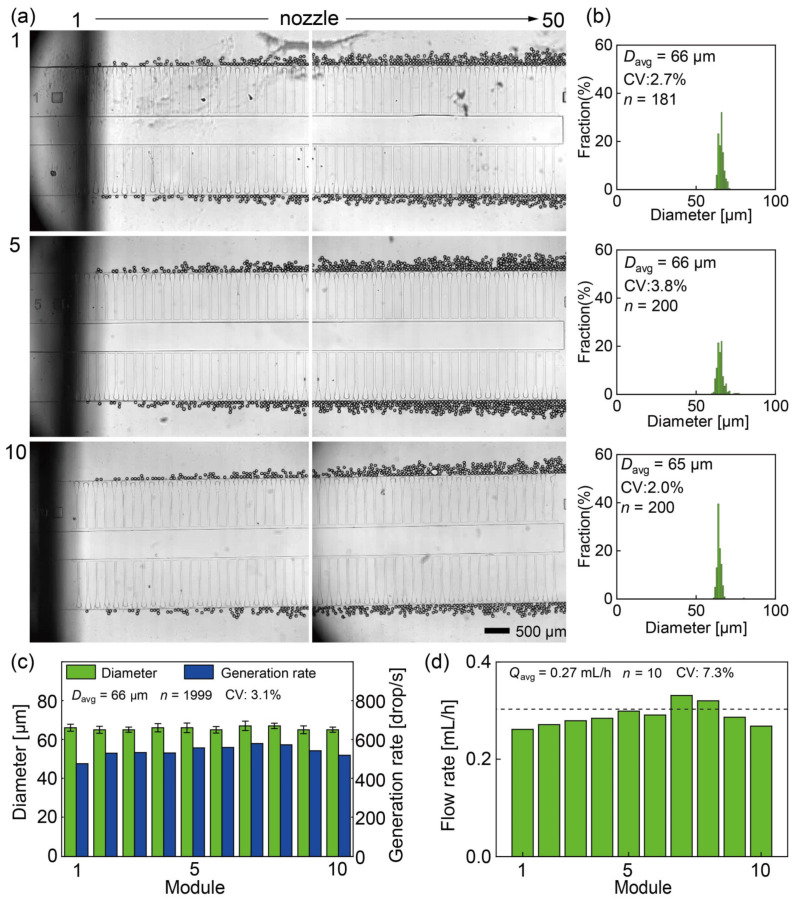
Step emulsification with the dispersed phase flow rate *Q*_d_ set at 3.0 and the continuous phase flow rate *Q*_c_ set at 50.0 mL/h. (**a**) Photomicrographs of the operating nozzles in modules #1, #5, and #10. (**b**) Size distributions of the main droplets in the three modules. (**c**) Analysis of diameters and generation rates of the main droplets in each module. (**d**) The calculated dispersed phase flow rates in each module.

Prior to the experiment, air was removed from the channels by priming the device with PVA solution. Upon fluid infusion into the inlet reservoirs, the HDDA filled the top part of the input reservoir before cascading downward into the ten modules, ensuring a stable fluid supply from the inlet reservoir to the microchannels. In particular, when the continuous phase flow rate (*Q*_c_) and dispersed phase flow rate (*Q*_d_) were maintained at 50.0 and 3.0 mL/h, respectively, uniform-sized droplets were formed in the dripping regime across each module (Figure 4a and Figure S3a, Video S1).

Among the 1000 step-emulsification nozzles distributed among the ten modules, 986 nozzles (98.6%) successfully produced droplets, whereas 14 nozzles (1.4%) experienced accidental clogging, resulting in the absence of droplet formation. The main droplet measurements revealed an average diameter of 66 μm with a CV of 3.1% (*n* = 1999) (Figure 4b,c and Figure S3b). Each module produced highly monodisperse main droplets, with average diameters of 66 ± 2, 65 ± 2, 65 ± 1, 66 ± 2, 66 ± 2, 65 ± 2, 67 ± 2, 67 ± 1, 65 ± 2, and 65 ± 1 μm. Notably, droplet coalescence was not observed around the nozzle. 

We quantified the droplet production rate (*F*) of the modules, with a mean production rate of 539.5 ± 28.6 drop/s (*n* = 10) per module, totaling 5395 drop/s for the entire device (Figure 4c). Subsequently, we calculated the throughput of the dispersed phase by multiplying the sizes of the main droplets by their production rates in each module (Figure 4d). While the flow rates in the outer modules tended to be lower and higher in the central modules, module 7 and module 8 exhibited unexpectedly higher flow rates compared to module 5 and module 6, potentially owing to fabrication errors. However, this variation was deemed insufficient to affect the sizes of the droplets generated across the ten modules because of the robustness of step emulsification against small flow rate variations.

**Figure 5:**
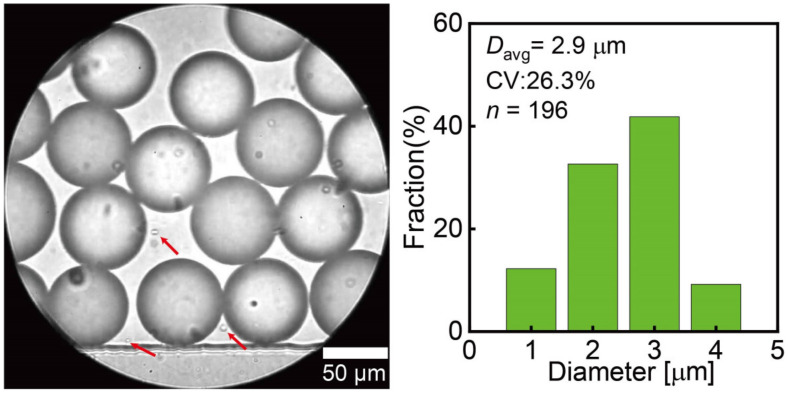
Satellite droplets (indicated by arrows) within the channel and their size distribution.

Besides the main droplets, satellite droplets were observed around the nozzles within the ten modules, having an average diameter of 2.9 μm and a CV of 26.3% (*n* = 196, Figure 5).

### 3.3. DLD Separation of Main and Satellite Droplets in Parallelized Modules

**Figure 6:**
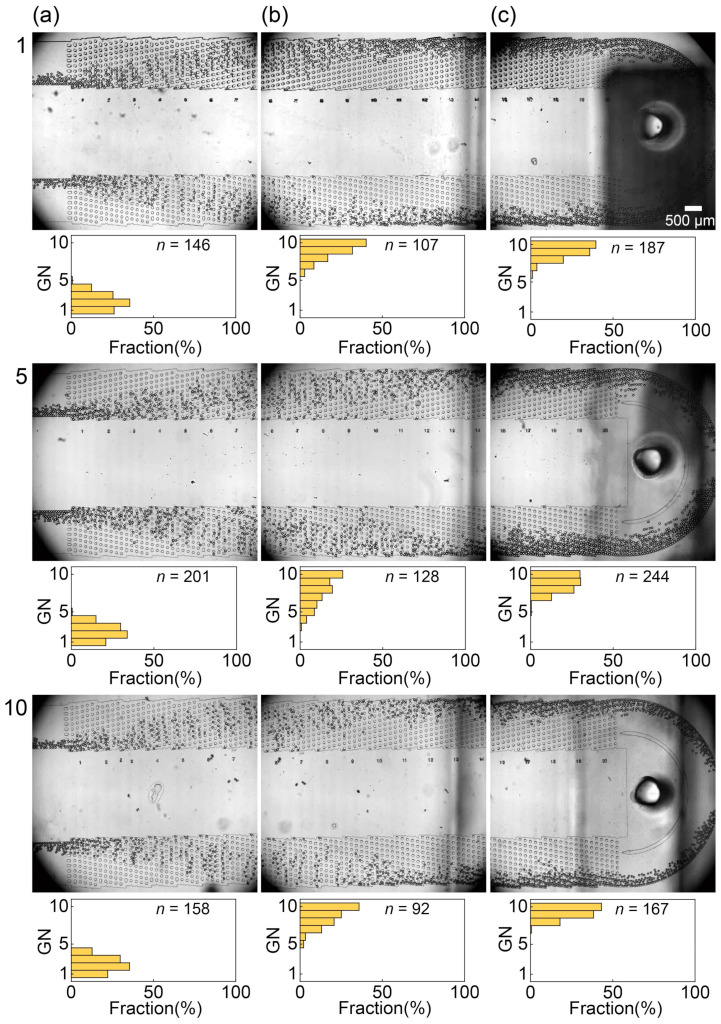
Spatial distribution of main droplets flowing through the DLD pillar arrays with *Q*_d_ set at 3.0 mL/h and *Q*_c_ set at 50.0 mL/h. (**a**) Main droplets entering the DLD region near the central wall in modules #1, #5, and #10. (**b**) Main droplets flowing through the midstream region in bump mode. (**c**) Displaced main droplets entering the outlet L.

In this section, we explored the migration patterns of both the main and satellite droplets within the parallel DLD regions based on their respective sizes. The migration behavior of the main droplets through the DLD micropillar arrays across all ten modules at flow rates of *Q*_c_ = 50.0 mL/h and *Q*_d_ = 3.0 mL/h is illustrated in Figure 6 and Figure S4 (Video S2). After being generated at the nozzles, the main droplets, with an average diameter of approximately 66 μm, predominantly flowed near the central wall of the nozzle array and entered the DLD region adjacent to the wall in all ten modules. At this flow rate, minimal droplet accumulation was observed at the entrance of the DLD, and no evidence of droplet deformation or coalescence within the modules was observed. 

After the main droplets began migrating toward the sidewall in the bump mode (Figure 6a and Figure S4a,d), they continued in this mode through a slightly increased number of gaps (gaps 3–10; Figure 6b and Figure S4b,e) in the midstream region toward the sidewalls. In the downstream region, further displacement occurred as the main droplets flowed through gaps 4–10 near the sidewall and they were eventually collected at outlet L (Figure 6c and Figure S4c,f). Approximately 60% of the droplets deviated from the expected path due to mutual collisions between closely flowing droplets, as observed in our previous study [26]. Additionally, a small fraction of the main droplets (0.4%) flowed through gaps 1–3 and were collected via outlet S, whereas the majority (99.6%) with a diameter larger than *D*_c_ (47 μm) traversed the pillars in bump mode, as anticipated. 

**Figure 7:**
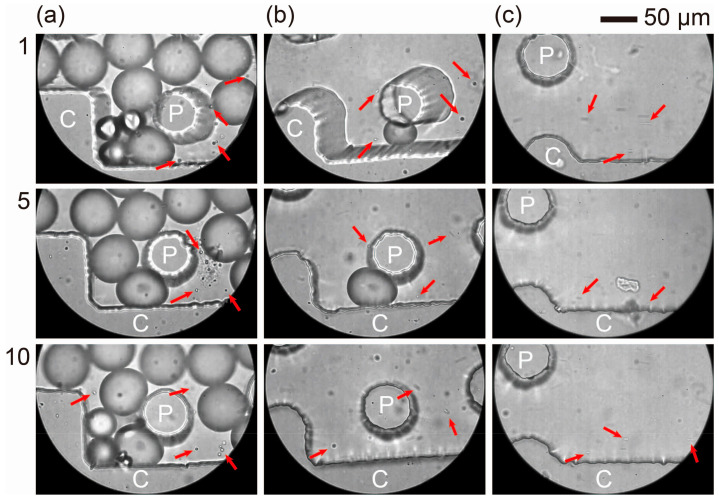
Satellite droplets flowing through the DLD pillars. (**a**–**c**) Satellite droplets (indicated by arrows) moving through the pillars in zigzag mode in the (**a**) upstream (DLD entrance), (**b**) midstream (11th DLD section), and (**c**) downstream (DLD exit) regions in modules #1, #5, and #10. Flow rates were *Q*_d_ = 3.0 mL/h and *Q*_c_ = 50.0 mL/h. The labels “C” and “P” denote the central wall and pillars in each module, respectively.

We also investigated the behavior of the satellite droplets in the ten DLD regions. Upon entering the DLD region near the central wall (gaps 1–2; Figure 7a and Figure S5a, Video S3), the satellite droplets maintained their vertical position with respect to the flow, exhibiting a zigzag mode as they moved near the central wall (gaps 1–2; Figure 7b and Figure S5b) in the midstream region. The satellite droplets continued to move near the central wall in the downstream region (gaps 1–2; Figure 7c and Figure S5c) and were finally collected via outlet S. 

**Figure 8:**
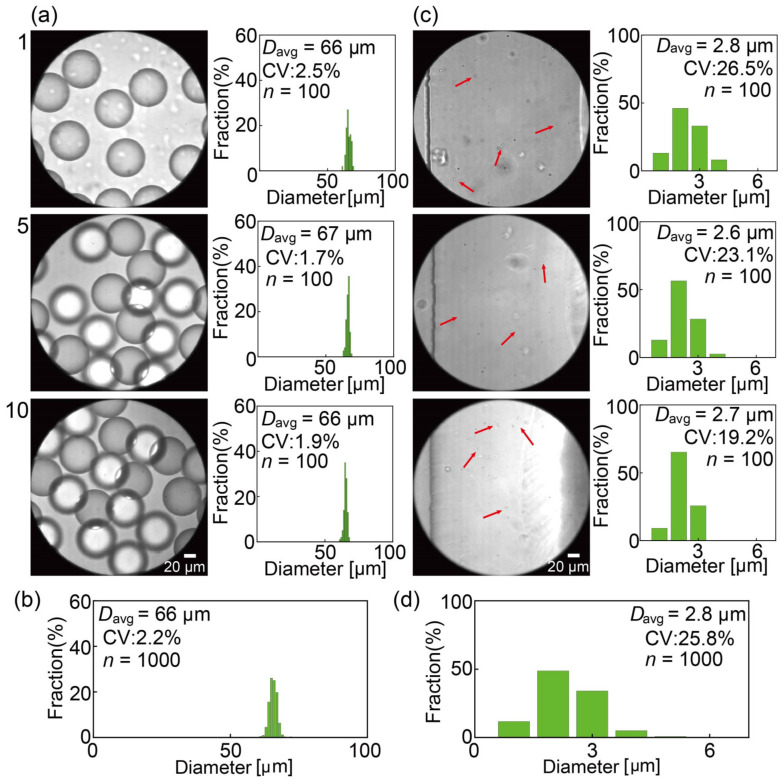
DLD separated droplets near the outlet reservoirs. (**a**) Main droplets flowing into outlet L with their respective size distributions in modules #1, #5, and #10. (**b**) Size distribution of the main droplets collected by outlet L. (**c**) Satellite droplets (indicated by arrows) flowing into outlet S and their respective size distributions in the three modules. (**d**) Size distribution of the satellite droplets collected by outlet S.

Following the successful separation of the main and satellite droplets within the DLD arrays, we observed these droplets at the outlets of all the modules and measured their sizes. The main droplets were exclusively observed at outlet L, whereas satellite droplets were exclusively observed at outlet S across all modules. The droplets collected at outlet L of the ten modules had an average diameter of 66 μm with a CV of 2.2%, which is consistent with the previously measured sizes of the main droplets (Figure 8a,b and Figure S6a). Similarly, the satellite droplets in outlet S had an average diameter of 2.8 μm with a CV of 25.8%, which is consistent with earlier measurements (Figure 8c,d and Figure S6b). This finding demonstrates the effective separation of the main and satellite droplets in all modules without significant volume loss, achieving the main droplets with a purity of 100%. Thus, in all parallel-arrayed modules with DLD pillars, satellite droplets with diameters smaller than *D*_c_ (47 μm) followed a distinct migration path from the main droplets, facilitating their efficient separation in the parallel-arrayed device with high throughput (3.0 mL/h).

The device demonstrated in this study builds upon our previous work by incorporating enhancements to an existing single module of 60 step-emulsification nozzles and DLD array for droplet production [26]. By incorporating 1000 step-emulsification droplet makers into a parallel array, this device achieved significantly higher generation rates, reaching 5395 drops/s and 3.0 mL/h. This result is a substantial improvement over the generation rate of the single step-emulsification-DLD module, which was limited to 408 drops/s and 0.2 mL/h. Compared to our previous study using the same materials, a 15-fold increase in throughput volume has been achieved. Additionally, unlike the eight previously reported, parallel, flow-focusing droplet makers and DLD arrays [25], the robustness of step emulsification against flow fluctuations enabled the parallelization of more nozzles within a single module and the incorporation of more modules within the same glass slide area (76 mm × 52 mm).

Although our device offers a higher throughput of satellite-free droplets, a small fraction of the main droplets (0.4%) deviated from the theoretical collection path owing to collisions between the densely packed droplets. To address this limitation, two approaches are considered. First, enlarging the bifurcated channel will be effective to facilitate the collection of main droplets that did not undergo sufficient displacement. Second, increasing the height of the DLD array to enhance its capacity will reduce the density of the droplets and mitigate collision-induced deviations. These strategies can further enhance the performance and versatility of the device for various droplet manipulation applications.

Unlike other large-scale systems designed for massive droplet production [29,30], our device aims to mass-produce “satellite-free” droplets, which is a distinct advantage. While the throughput of our current device is lower, the size uniformity of the main droplets is similar to non-satellite-free systems. We believe the throughput can be significantly increased by incorporating more nozzles and modules in a device with a larger footprint. Additionally, stacking and/or parallelizing such devices presents a promising approach for the large-scale production of satellite-free droplets. Finally, as demonstrated in previous systems, our device can be made from solvent-resistant materials like glass and silicon, which may be more suitable for maintenance and long-term operation in industry. Meanwhile, the PDMS-based device used in this study is cost-effective and suitable for laboratory-scale testing as a nearly disposable device.

## 4. Conclusions

In this study, we introduced a novel approach to enhance the production and yield of satellite-free droplets by implementing a parallel setup that integrated serially arranged step emulsification and DLD for droplet generation and separation. We ensured a uniform fluid supply using the inlet reservoirs of the top layer, which facilitated droplet generation rates across all ten modules. Separation in the DLD arrays within these modules captured monodisperse main droplets, characterized by an average diameter of approximately 66 μm, a CV of 2.2%, a purity of 100%, and a recovery rate of 99.6%. Moreover, our method significantly increased the yield of satellite-free droplets, achieving a production rate of 3.0 mL/h. Consequently, a substantial improvement was observed compared to our previously reported production using a single-module device (0.2 mL/h). The high yield capability and contamination-free features of our approach makes it promising for use in various industrial production settings.

## Data Availability

The data presented in this study are available from the corresponding author on reasonable request.

## Supplementary Materials

The following supporting information can be downloaded at: https://www.mdpi.com/article/10.3390/mi15070908/s1, Figure S1: Step-emulsifier nozzles made from poly(dimethylsiloxane); Figure S2: Model of the hydraulic resistance of the inlet and outlet reservoirs as well as the step-emulsification and DLD modules; Figure S3: Step emulsification with the dispersed phase flow rate *Q*_d_ set at 3.0 and the continuous phase flow rate *Q*_c_ set at 50.0 mL/h; Figure S4: Spatial distribution of main droplets flowing through the DLD pillar arrays with *Q*_d_ set at 3.0 mL/h and *Q*_c_ set at 50.0 mL/h; Figure S5: Satellite droplets flowing through the DLD pillars; Figure S6: DLD separated droplets near the outlet reservoirs; Table S1: Flow rate ratios calculated by Equation (3); Video S1: Step emulsification in modules #1, #5, and #10; Video S2: Main droplet migration through the DLD pillars; Video S3: Satellite droplet migration through the DLD pillars.
