# Supplementary material for: Upscaled Production of Satellite-Free Droplets: Step Emulsification with Deterministic Lateral Displacement
Source: Micromachines (Basel). 2024 Jul 12;15(7):908. doi: 10.3390/mi15070908 (PMC11278866; doi:10.3390/mi15070908)
Supplement: Supplementary file 1 [file micromachines-15-00908-s001.zip › Suppl_TN240620.pdf]

Supplementary Material

Upscaled Production of Satellite-Free Droplets: Step Emulsification with Deterministic Lateral Displacement

Guangchong Ji[1], Shuzo Masui[2], Yusuke Kanno[2] and Takasi Nisisako[2,*]

[1] Department of Mechanical Engineering, School of Engineering, Tokyo Institute of Technology, Tokyo 152-8550, Japan; ji.g.aa@m.titech.ac.jp

[2] Institute of Innovative Research, Tokyo Institute of Technology, R2-9, 4259 Nagatsuta-cho, Midori-ku, Yokohama, Kanagawa 226-8503, Japan; masui.s@nanolab.t.u-tokyo.ac.jp (S.M.); kanno.y.ag@m.titech.ac.jp (Y.K.)

* Correspondence: nisisako.t.aa@m.titech.ac.jp

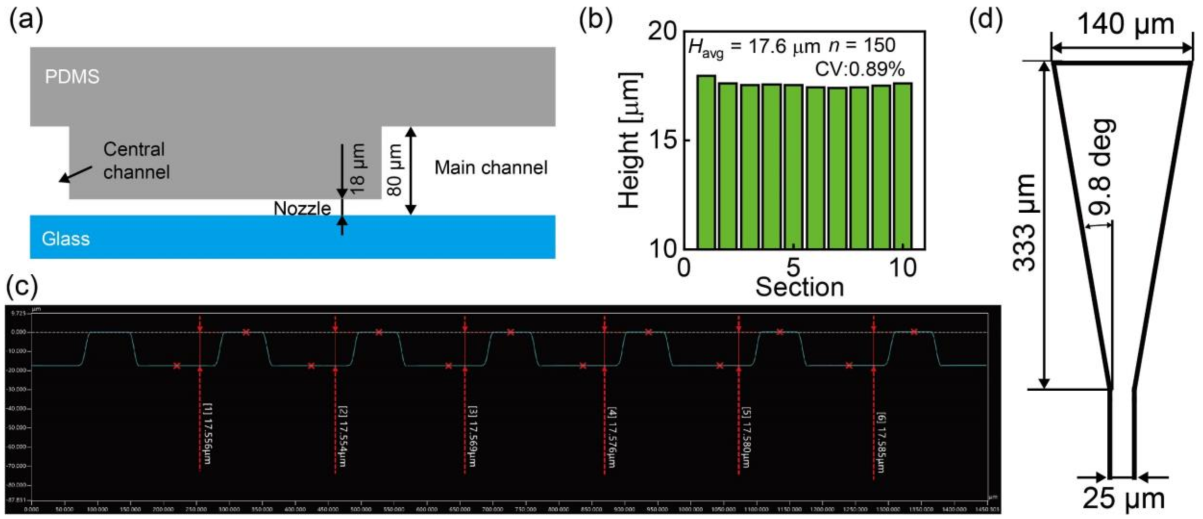

Figure S1. Step emulsifier nozzles made from poly(dimethylsiloxane) (PDMS). (a) Schematic illustration of the cross-section of a step-emulsifier nozzle (b) Height distribution of the PDMS step-emulsifier nozzles across 10 modules. (c) Dimensions of the triangular end of the step-emulsifier nozzle. (d) Heights of a subset of the PDMS step emulsifier nozzles measured with a laser microscope (VK-X3000; Keyence, Osaka, Japan).

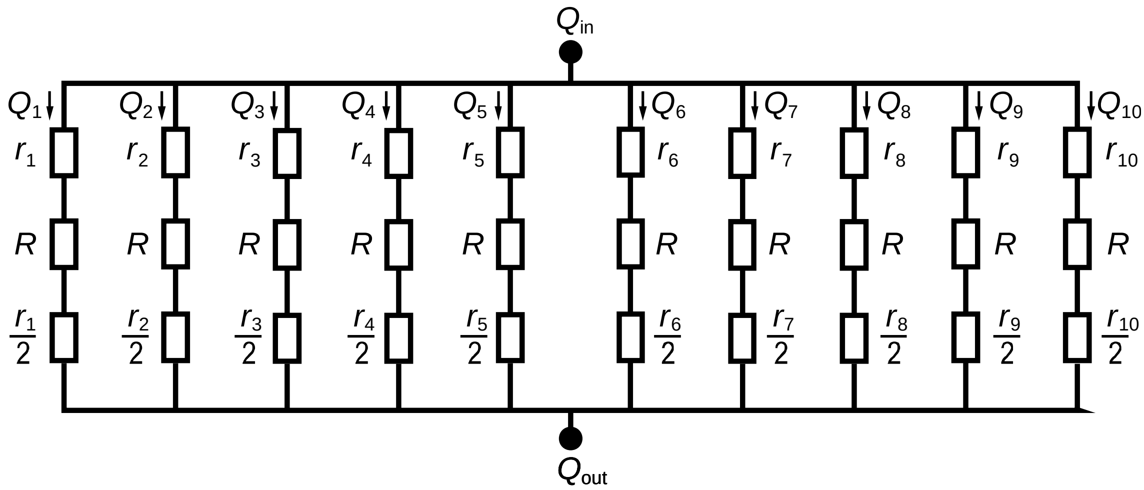

Figure S2. Model of the hydraulic resistance of the inlet and outlet reservoirs as well as the step-emulsification and DLD modules. R represents the resistance of a module in the lower section (= $0.5R_c + 0.5R_D$), r denotes the resistance of the inlet and outlet chambers, and Q indicates the flow rate in a module.

Table S1. Flow rate ratios calculated by equation (3)

Module #	100 * Q_n/Q_1 (%)	Module #	100 * Q_n/Q_1 (%)
1	100	6	100.06
2	100.01	7	100.03
3	100.01	8	100.02
4	100.03	9	100.01
5	100.06	10	100.00

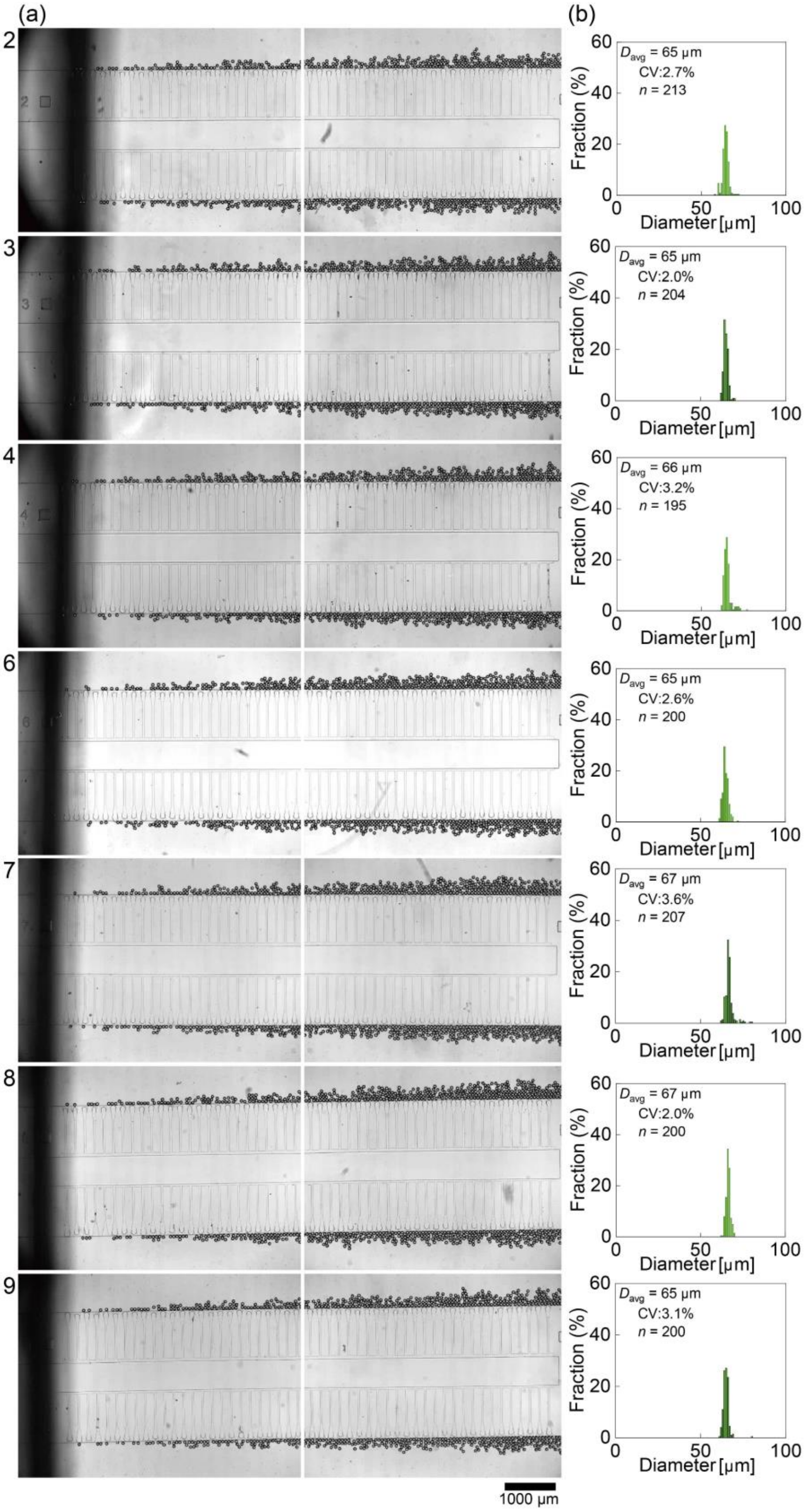

Figure S3. Step emulsification with the dispersed phase flow rate Q_d set at 3.0 and the continuous phase flow rate Q_c set at 50.0 mL/h. (a) Photomicrographs of the operating nozzles in seven modules (excluding #1, #5, and #10). (b) Size distributions of the main droplets.

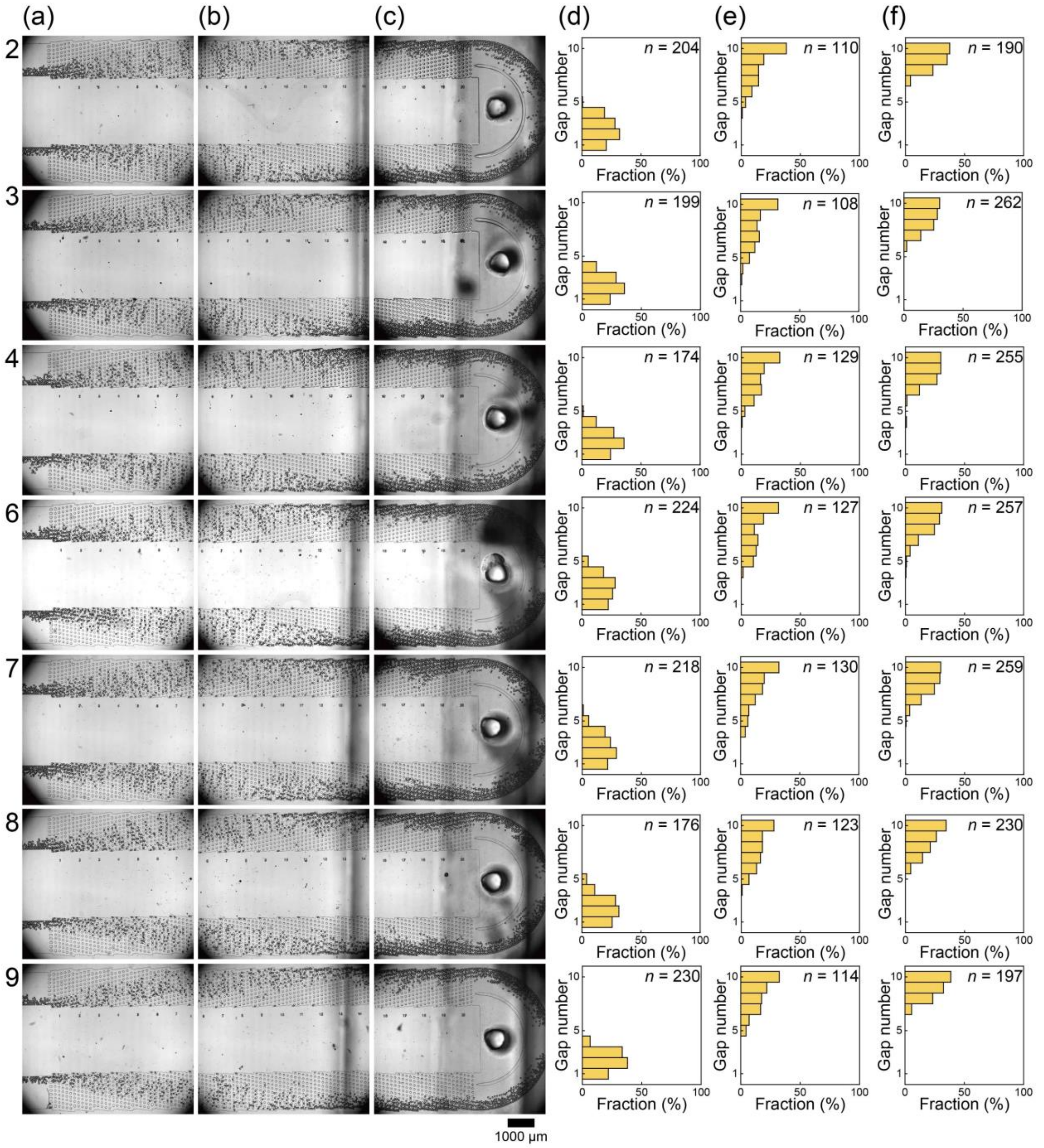

Figure S4. Spatial distribution of main droplets flowing through the DLD pillar arrays with Q_d set at 3.0 mL/h and Q_c set at 50.0 mL/h. (a) Main droplets entering the DLD region near the central wall in seven modules (excluding #1, #5, and #10). (b) Main droplets flowing through the midstream region in bump mode. (c) Displaced main droplets entering outlet L. (d-f) Spatial distribution of the main droplets in the (d) upstream, (e) midstream, and (f) downstream regions of the DLD arrays.

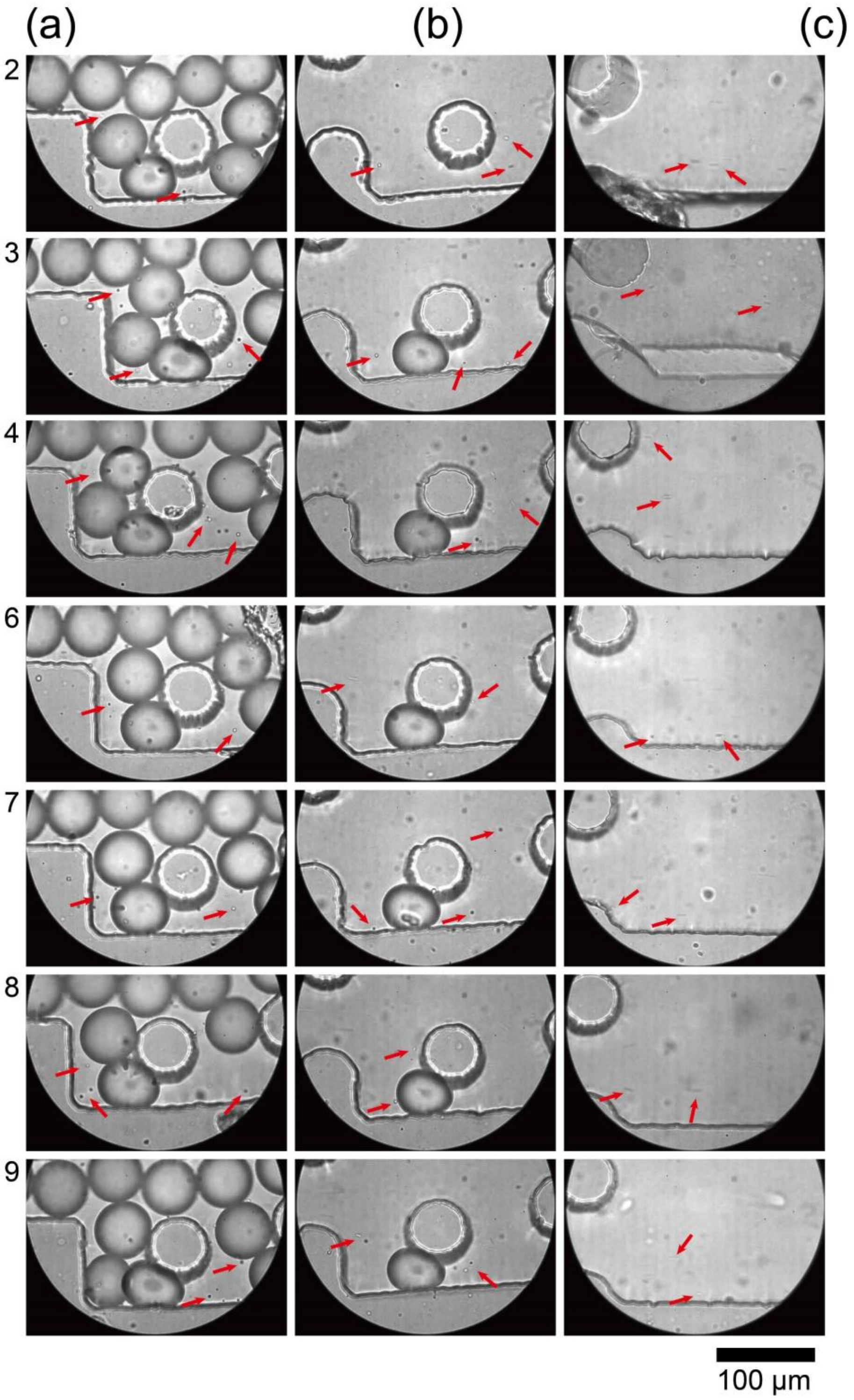

Figure S5. Satellite droplets flowing through the DLD pillars. (a-c) Satellite droplets (denoted by arrows) moving through the pillars in zigzag mode in the (a) upstream, (b) midstream, and (c) downstream regions in seven modules (excluding #1, #5, and #10). Flow rates were Q_d = 3.0 mL/h and Q_c = 50.0 mL/h.

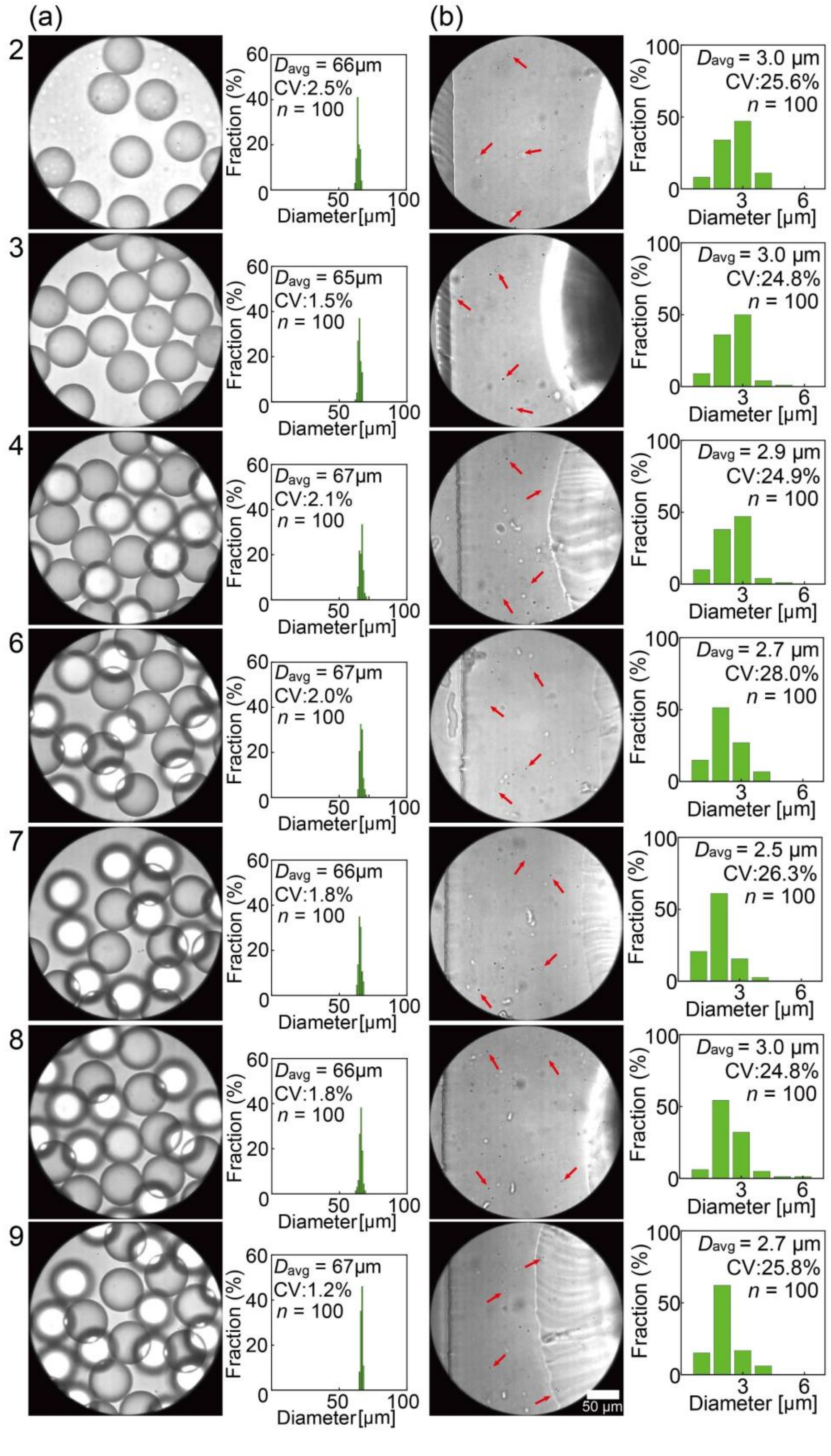

Figure S6. DLD separated droplets near the outlet reservoirs. (a) Main droplets flowing into outlet L and their size distributions in seven modules (excluding #1, #5, and #10). (b) Satellite droplets (denoted by arrows) flowing into outlet S and their size distributions.

Supplementary Movie Captions

Video S1: Movie clip of step emulsification in modules #1, #5, and #10, recorded at 50 fps.

Video S2: Movie clip of the migration of main droplets in the (a) upstream, (b) midstream, and (c) downstream regions of the DLD micropillar array in modules #1, #5, and #10, recorded at 50 fps.

Video S3: Movie clip of the migration of satellite droplets in the (a) upstream, (b) midstream, and (c) downstream region of the DLD micropillar arrays in modules #1, #5, and #10, recorded at 500 fps.

Flow rates of the disperse phase and continuous phase in all videos were 3.0 mL/h and 50.0 mL/h, respectively.

All files are in MPEG-4 format.
